# Physician’s Perception Toward Using Telemedicine During COVID-19 Pandemic in King Abdulaziz Medical City, Riyadh, Saudi Arabia

**DOI:** 10.7759/cureus.16107

**Published:** 2021-07-02

**Authors:** Bader A Altulaihi, Khalid G Alharbi, Abdulrahman M Alhassan, Abdullah M Altamimi, Mouneera A Al Akeel

**Affiliations:** 1 Family and Community Medicine, King Abdulaziz Medical City, Riyadh, SAU; 2 Family Medicine, King Saud bin Abdulaziz University for Health Sciences, Riyadh, SAU; 3 Family Medicine, King Saud Bin Abdulaziz University for Health Sciences, Riyadh, SAU; 4 Pharmacy, Ministry of Health, Riyadh, SAU

**Keywords:** saudi arabia, covid-19, virtual clinics, physician perception, telemedicine

## Abstract

Introduction

The novel coronavirus, officially known as COVID-19, was first reported in Wuhan, China, in December of 2019. Since that time, medical services in Saudi Arabia have adapted to the situation by delivering medical care via virtual clinics. Therefore, the aim of the study is to assess physicians' perception and the level of satisfaction with telemedicine during the COVID-19 pandemic in Riyadh, Saudi Arabia.

Methods

This was a cross-sectional study that included family medicine consultants and fellows who had used telemedicine in primary health care centers in Riyadh, Saudi Arabia. It was conducted using an online validated questionnaire. The questionnaire was completed by 219 family medicine consultants and fellows, after obtaining their informed consent. The data that were extracted from the questionnaire included demographics, level of satisfaction, and questions related to their experience with telemedicine.

Results

Two hundred and nineteen participants enrolled in this study with 50.6% males and 49.4% females. The overall level of physicians’ satisfaction with telemedicine was 64.3%. However, only one-third preferred telemedicine over office visits. Of these, 60% were males and 40% were females. The only factor that had a statistically significant effect on the preference of office visits or telemedicine was time efficiency (p-value < 0.001). Of those who preferred office visits over telemedicine, 52% of them cited ease of discussion and the ability to make a comprehensive physical examination as the most important reasons for choosing office visits. Technologic issues were the least important factor for choosing either clinic (4.1%). Of those who preferred telemedicine, avoiding contact with patients suspected of COVID-19 was the most commonly cited factor (27.4%). Family medicine physicians face multiple barriers while using telemedicine during the COVID-19 pandemic. The most commonly cited barrier was the inability to make a full and comprehensive assessment of the patient.

Conclusions

In the setting of highly transmissible disease epidemics, telemedicine has a lot of potential for providing quick and safe care that is appropriate for screening and management. Based on our findings, using telemedicine should be encouraged by improving physicians’ skills in this field since telemedicine is a crucial step to reduce the risk of COVID-19 transmission and provide community-wide treatment.

## Introduction

COVID-19, the official name of the novel coronavirus, was initially reported in Wuhan, China, in December of 2019 [1]. Since then, it has spread all across the globe. Currently, it has affected over 175 million individuals in 210 countries [2].

As of June 10, 2021, nearly half a million COVID-19 cases have been confirmed in Saudi Arabia with a death toll of more than 7,000 [2]. The first confirmed case of the novel coronavirus in Saudi Arabia was on March 2, 2020 [3].

In the era of COVID-19, regular outpatient clinics pose a great health risk for both the clinician and the visiting patient [4]. Therefore, an urgent need emerged to switch health care provision from physical contact in outpatient clinics to addressing patients’ complaints through telemedicine [5]. Telemedicine is defined as the usage of electronic communication technologies to provide health care beyond patients’ distance [6]. Telemedicine gives the opportunity for patients and clinicians to have regular appointments and visits through the phone or video call without the risk of virus spread [7].

Physicians’ perception of telemedicine is an important component for the success of telemedicine. Nevertheless, there are some factors that govern how physicians perceive telemedicine service. Knowledge, technical training, and the presence of clear guidelines and standards for appropriate application are necessary for physicians to uptake telemedicine [8]. Indria et al. [9] aimed to assess clinicians’ perception of telemedicine. Physicians in this study agreed that telemedicine provided quicker access to health care, but participants stated that poor communication technology was a barrier to having a better consultation outcome.

Telemedicine has always been available even before the COVID-19 pandemic. In 2015, a Cochrane systematic review [10] examined the difference between telemedicine and in-person visits for the management of chronic diseases such as diabetes mellitus and heart failure. Results showed no difference in health outcomes between in-person visits and telemedicine. Another study was done in the United States [11] aiming to assess how patients perceive telemedicine through primary care video visits. Authors have reported that the majority were interested in continuing video calls over in-person visits. This finding is crucial, nowadays, given the spread of the novel coronavirus.

COVID-19 pandemic is changing telemedicine outlook with rapid speed. A study done in India [12] showed that telemedicine service was feasible and acceptable even in rural areas. On the other hand, a study conducted in the United States [13] concluded that there was uncertainty regarding the effectiveness and feasibility of telemedicine. Up to our knowledge, the perception of health care workers toward telemedicine has not been rigorously assessed locally. Therefore, the aim of this study was to assess the barriers, benefits, and physicians' perceptions of telemedicine in the primary health care (PHC) center in Riyadh, Saudi Arabia.

## Materials and methods

This was a cross-sectional study that included 219 family medicine consultants and fellows who used telemedicine in any of the three main PHC centers in National Guards Health Affairs, Riyadh, Saudi Arabia. These PHC were Khashm-Ala’an, Umm-alhammam, and Iskan centers. This study was approved by the International Review Boards of King Abdullah International Medical Research Center (KAIMRC), Ministry of National Guard - Health Affairs, Riyadh, Kingdom of Saudi Arabia (approval number NRC21R/077/02). The inclusion criteria were all family medicine consultants and/or fellows who had experienced telemedicine in National Guards PHC centers. The study's population was estimated to be approximately 300 physicians, which was the total number of family medicine consultants and fellows working in National Guards PHC centers. Our sample size was trying to get as close to 300 responses as possible with a confidence interval of 95%. The sampling technique used in this study was a convenient sampling technique in which all eligible participants were included. The study was conducted using an online questionnaire distributed to the participants through work e-mails. The double response was prevented by allowing participants to have one response only. This was checked by having the participants log into their e-mail accounts to confirm they have not answered the questionnaire previously. The questionnaire was distributed to the participants in the English language as all participants are health care workers. Then the questionnaire was piloted by sending it to 30 participants, of which 25 responses were retrieved. It was then distributed formally to 300 participants. Data collected in the questionnaire included demographic data, quality of virtual clinic service, 11 questions related to telemedicine experience, and two questions on physicians’ attitude toward telemedicine, and how it compared to regular office visits. The level of significance was set at a p-value of <0.05, and data analysis was performed using the Statistical Package for Social Science (SPSS) version 24.0 (IBM Corp., Armonk, NY, USA).

## Results

Two hundred and nineteen respondents completed the survey with a response rate of 73%. Table 1 shows the number and percentages of participants’ demographic data. Overall, 111 of the participants were males (50.6%) and 108 were females (49.4%).

**Table 1:** The number and percentages of participants’ demographic data N = Number % = percentage

	N	%
Gender		
Male	111	50.6
Female	108	49.4
Marital Status		
Married	140	63.9
Unmarried	79	36.1

Physicians included in our study were asked about their clinic preferences. Nearly two-thirds (59.8%) of the respondents preferred office visits as compared to those who opted to choose telemedicine clinics (28.3%). The remaining one-fifth of the participants did not think that there was a difference between the two clinics. Of those who prefer telemedicine, 60% were males while the rest were females (40%).

**Figure 1:**
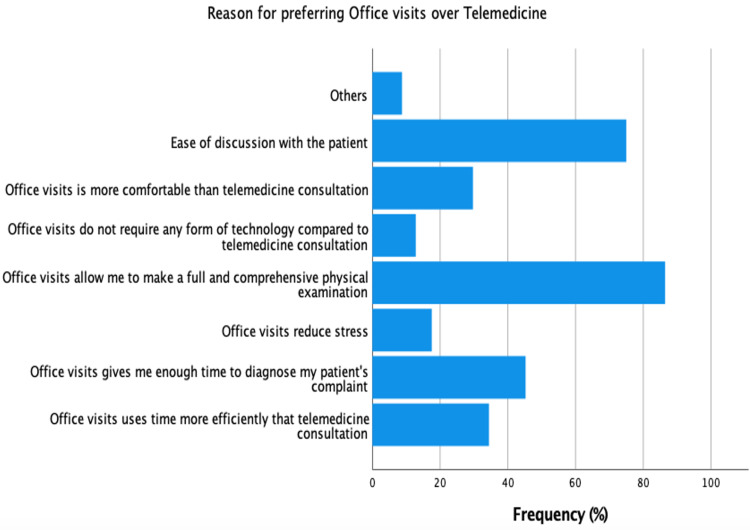
Reasons for preferring office visits over telemedicine

**Figure 2:**
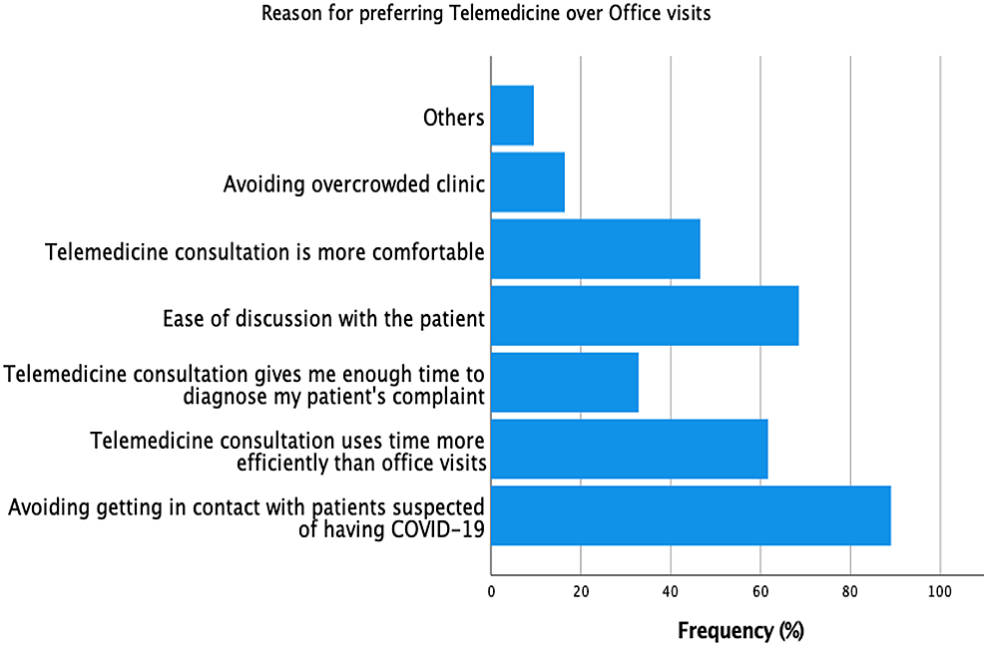
Reasons for preferring telemedicine over office visits

The study’s participants had varying reasons for choosing their clinic preference. Figure 1 shows the reasons for preferring office visits, while Figure 2 shows the reasons for preferring telemedicine. Of those who preferred office visits, 52% of them cited ease of discussion and the ability to make a comprehensive physical examination as the most important reasons for choosing office visits over telemedicine. Technologic issues were the least important factor for choosing either clinic (4.1%). Of those who preferred telemedicine, avoiding contact with patients suspected of COVID-19 was the most commonly cited factor (27.4%).

When gender was cross-tabulated with the factors governing clinic preference, males were more concerned about the comfort of either clinic than females (65% vs 35%). Females were more concerned about the level of stress in each clinic and time efficiency compared to males.

Even though males and females had different clinic preferences, gender did not have a statistically significant effect on the preference of either telemedicine or office visits (p-value = 0.348). Similarly, privacy concerns and technological training did not significantly affect clinic preference. Time efficiency, however, was the only significant factor affecting the choice of the clinic (p-value < 0.001).

When respondents were asked whether they were satisfied with telemedicine in terms of diagnosing patients’ complaints, the majority (60.6%) were not satisfied with their ability to diagnose their patients’ complaints using telemedicine. Furthermore, when patients were treated, 58% of the respondents thought that the therapeutic effectiveness and compliance might not increase if the treatment was prescribed through telemedicine as compared to the office visits.

Many of our participants (75%) had some barriers while using telemedicine. The most commonly faced barrier was the inability to make a full and comprehensive assessment of the patient. The least cited barrier was distraction faced from the patients’ side/setting.

When respondents compared telemedicine and office visits, nearly half of them thought that telemedicine took less time to assess and treat patients’ complaints compared to office visits. However, in terms of the efficiency of the consultation, 70% of the family physicians included in our study perceived office visits as being more efficient than telemedicine.

We asked our participants about their thought on the ability to fully perform the first consultation using telemedicine, the majority of them (54%) claimed that first visits cannot be done through telemedicine. On the other hand, follow-up appointments were more accepted by our participants to be done through telemedicine as 64.3% of the physician included thought that the patient was always available when called for follow-up appointments through telemedicine.

## Discussion

This study aimed to assess physicians’ perception, benefits, and barriers toward telemedicine during the COVID-19 pandemic. 71.7% of the participants favored office visits, while 28.3% favored telemedicine over in-office appointments or clinics. Of those, males were more likely to prefer telemedicine. In comparison to physicians preferring telemedicine, there were no gender differences among physicians favoring office clinics. However, we had not been able to find any statistical significance in the effect of gender on the preference of telemedicine or office clinics (p-value = 0.348).

Different studies delved into physicians’ perceptions toward telemedicine, one of which showed that ER physicians were the least likely to prefer telemedicine [14], while 96% of radiation oncologists foresee telemedicine to have a role beyond the pandemic [15]. Furthermore, 80% of ophthalmology patients preferred to have telemedicine as an option for first-visit evaluation [16]. The diversities of preferences could be related to the nature of the specialty and the physician's capability to deliver health care.

In this study, we found that fear of contacting patients with possible COVID-19 patients was the most commonly cited reason among those who prefer telemedicine. Ease with discussion was the second reason, and efficient time using with telemedicine was the third. Of those three, time spent during a consultation had a statistically significant effect on the preference of telemedicine over office clinics (p-value = 0.001). Some studies suggested that technical issues, feasibility of telemedicine, and privacy and confidentiality issues were additional significant barriers in applying telemedicine [17,18]. Nevertheless, we could not find a statistical significance in the effect of technology needed for telemedicine (p-value = 0.084) and concerns of privacy while using telemedicine (p-value = 0.111). The earlier p-value of the effect of technology on the preference of telemedicine, however, should be taken into special consideration. This is because our sample size (n = 219) was not very high and if our study were to be replicated in a similar setting but with a higher sample size, the p-value could get <0.05 because of its proximity to this cut-off point and, hence, be significant. This is especially true if telemedicine practiced in Saudi Arabia were to include more advanced technologies such as video-assisted telemedicine.

Similar to other studies, physicians favored office clinics because of the need to make comprehensive assessments including physical examinations [14,19]. In-office appointments allowed the physicians to do a full assessment, admit the patient if needed, and provide acute care. Heyer et al. clarified that it was difficult to instruct the patients to do a physical examination, and physical examination that was done through telemedicine was limited to inspection most of the time [19]. Despite that, the vast majority of this study’s participants agreed that contacting patients using telemedicine was easy, and the information provided to the physician was clear, 61% of our participants believed that taking history using telemedicine was not easier than office clinics. In fact, more than half of the participants were convinced that telemedicine was not sufficient for diagnosis. These barriers could be attributed to language/cultural differences, speech/comprehension problems among patients, and lack of equipment or skills to use telemedicine, especially among elderly patients. On the other hand, well-designed studies showed that telemedicine decreased wait times, missed appointments, readmissions, and improved treatment compliance and clinical outcomes all while minimizing the cost on the patients and the health system [20-22].

Half of our participants had described their telemedicine experience to be convenient and satisfactory. Likewise, Indria et al. had noted that the satisfaction rate with telemedicine among their participants was high [11]. In contrast, Coutrney et al. demonstrated that 80% of the neurologist had a reduction in work satisfaction when using telemedicine [23]. This difference could be attributed to the difference in technology used by neurology telemedicine consultations as compared to primary care or family physician telemedicine consultations. The earlier might use more video-assisted telemedicine consultation technology because of the robust physical exam required by these specialists.

Several limitations were found in this study. This study included only PHC physicians. Therefore, our findings may not be generalizable to represent the total satisfaction and quality of telemedicine in other specialties. The measurements of privacy and confidentiality were purely subjective to the physicians’ opinion. Different challenges were not addressed in the study such as cost-effectiveness and availability of appropriate infrastructure to practice telemedicine in all PHC centers. Telemedicine is one of the options that can bring health care closer to the patients. Therefore, with appropriate infrastructure, our findings may promise that a well-formed telemedicine system can decrease the load on the hospitals and provide high-quality health care with good outcomes.

## Conclusions

In the setting of highly transmissible disease epidemics, telemedicine has a lot of potential for providing quick and safe health care that is appropriate for screening and management. Most of the physicians in our study preferred to see the patients in person due to the inability to do a physical examination and full assessment by telemedicine method. This might be explained by the fact that the majority of the appointment in the three included PHC centers were new, which necessitates full assessment and physical examination. In addition, telemedicine is a relatively new experience for the physicians working in National Guards. As the COVID-19 epidemic spreads globally and based on our findings, using telemedicine should be encouraged by improving physicians’ skills in this field as the only solution to reduce the risk of COVID-19 transmission, and finally, provide community-wide treatment.
